# Prototype Generation Using Self-Organizing Maps for Informativeness-Based Classifier

**DOI:** 10.1155/2017/4263064

**Published:** 2017-07-25

**Authors:** Leandro Juvêncio Moreira, Leandro A. Silva

**Affiliations:** ^1^Graduate Program in Electrical Engineering and Computing, Mackenzie Presbyterian University, Sao Paulo, SP, Brazil; ^2^Computing and Informatics Faculty & Graduate Program in Electrical Engineering and Computing, Mackenzie Presbyterian University, Sao Paulo, SP, Brazil

## Abstract

The *k* nearest neighbor is one of the most important and simple procedures for data classification task. The *k*NN, as it is called, requires only two parameters: the number of *k* and a similarity measure. However, the algorithm has some weaknesses that make it impossible to be used in real problems. Since the algorithm has no model, an exhaustive comparison of the object in classification analysis and all training dataset is necessary. Another weakness is the optimal choice of *k* parameter when the object analyzed is in an overlap region. To mitigate theses negative aspects, in this work, a hybrid algorithm is proposed which uses the Self-Organizing Maps (SOM) artificial neural network and a classifier that uses similarity measure based on information. Since SOM has the properties of vector quantization, it is used as a Prototype Generation approach to select a reduced training dataset for the classification approach based on the nearest neighbor rule with informativeness measure, named *i*NN. The SOM*i*NN combination was exhaustively experimented and the results show that the proposed approach presents important accuracy in databases where the border region does not have the object classes well defined.

## 1. Introduction

The main task of a data classifier is to predict the class of an object that is under analysis. The simplest procedure for data classification tasks is the *k* nearest neighbor (*k*NN) algorithm. The algorithm strategy for classification comprises three operations: (i) an unlabeled sample is compared to dataset training through a similarity measure; (ii) the labeled objects are sorted in order of similarity to the unlabeled sample; and finally, (iii) the classification occurs giving the unlabeled sample the majority class of the nearest neighbors objects. Because of its simplified algorithm (three basic operations steps), and reduced number of parameters (similarity measure and the *k* number of nearest neighbor), this instance-based learning algorithm is widely used in the data mining community as a benchmarking algorithm [1–5].

Since the *k*NN algorithm has no model, an exhaustive comparison of the unlabeled sample with all the labeled and stored objects in the database is necessary, which increases the computational time of the process. In addition to this weakness of algorithm, the decision boundaries are defined by the instances stored in the training set and, for this, the algorithm has low tolerance to noise; that is, all training dataset objects are considered relevant patterns. Finally, the optimal choice of *k* depends upon the dataset mainly when the object analyzed is in a boundary region, making this parameter to be tuned according to the application [6–9].

To overcome the drawbacks above, there are in the literature different approaches such as similarity measure alternative to the Euclidean distance to minimize misclassification in boundaries region [10], methods to avoid searching the whole space of training set [11], and dataset summarization to find representative objects of training set [9]. For the dataset summarization approach, there are two main strategies to reduce the dataset volume: one of them based on instance selection and the other based on prototypes. For the approaches based on pattern (or instance) selection, the aim is to find a representative and reduced set of objects from the training dataset, which has the same or higher classification accuracy of a raw dataset [8, 12–15]. The strategies based on prototype, on the other hand, are defined in two approaches: Prototype Selection (PS) [16] and Prototype Generation (PG) [13, 17–19]. The approaches are equivalent; both can be used to identify an optimal subset of representative prototypes, discarding noise, and redundancy. The difference is that PG can also be used to generate and to replace the raw dataset by an artificial dataset. The use of prototypes or reduced training objects that are represented by prototypes minimizes some of *k*NN drawbacks previously mentioned as the exhaustive comparison of all training dataset.

Silva and Del-Moral-Hernandez [5] presented combination methods that use the winning neuron and topological maintain concepts of the Self-Organizing Maps (SOM) neural network to define a reduced subset of objects of the training set that are highly similar to the object that is under analysis for classification [5, 20]. This object subset is retrieved and then utilized by the *k*NN to execute the classification task. In other words, the SOM executes a preprocessing for the *k*NN classifier, recovering the similar objects from the winning neuron and from the adjacent neighbors of the SOM map [21].

With respect to drawback in the tuning of parameter *k*, Zhang et al. proposed a computation learning for this parameter [22]. Song et al., on the other hand, proposed a metric based on informativeness to perform the classification process in a boundaries region, where the choice of *k* is more sensible [10]. This algorithm was called *i*NN and the main idea is investigating the nearest objects more informative instead of the closest. This approach outperforms the use of *k*NN with Euclidean distance; however, it further increases the complexity of the comparison, consequently increasing process time [23].

Inspired by use of PG [5, 20, 21], we introduce a hybrid approach, where in a first step there is the SOM, which has the quantization vector and topological maintenance as important features for using it as a preprocessing in order to present to the classifier algorithm a reduced set of objects, highly similar to the unknown object that is being investigated. Next, the *i*NN algorithm will attribute a class to the unknown object based on the most informative objects of selected set. For the initial exploratory experiments, we observed important results of accuracy and time in classification process [23].

We here formally detail how SOM*i*NN works in hybrid architecture for classification problems. Besides that, here we introduced an experimental methodology to analyze qualitatively the SOM*i*NN classifier in three artificial datasets, experimenting different distribution in the region of class overlapping. In addition, we perform the experiments in 21 databases publicly (7 times more than in the previous study) available in the UCI repository and also sampling way by the 5-fold cross validation method in the complementary website to the paper published by Triguero et al. [9]. The results are analyzed using accuracy, kappa, prototype reduction, and time as performance indices.

The rest of the paper is organized as follows: in Section 2, a brief explanation of Prototype Generation and the taxonomy proposed by [9] are shown; Self-Organizing Maps and the methods to use them in classification with *k*NN are presented in Section 3. In Section 4, the experimental methodology is introduced. Experimental results, discussion, and comparative results are given in Section 5. In the last section, the conclusions are provided.

## 2. Theoretical Fundamental

### 2.1. A Brief Introduction to Prototype Generation

For a better understanding of the Prototype Generation idea, let us consider an object **x**_*n*_ of a dataset, defined as a set of descriptive attributes of *m* dimensional and with a class attribute *y*; that is, **x**_*n*_ = [*x*_*n*1_, *x*_*n*2_,…, *x*_*nm*_, *x*_*ny*_]. Then, let us assume that **X**_train_ is a training dataset with *N*_train_ samples of **x**_*n*_. The purpose of Prototype Generation (PG) is to obtain a reduced set, **X**_red_, with *N*_red_ instances selected or generated from **X**_train_, but with *N*_red_ ≪ *N*_train_. The cardinality of this reduced set must be sufficiently small to decrease the evaluation time taken by a classifier (*k*NN, for example), maintaining the classification accuracy. In fact, data reduction approaches aim mainly to summarize the raw dataset, without damaging the analytical properties, which implies performance accuracy.

For the PG methods, prototypes are used by classifiers instead of raw datasets, or they are used to generate an artificial dataset. Data generation can be interesting in some cases to eliminate data noise or to solve dataset with unbalanced class. Since the possibilities of usage are diversified, the literature presents different methods, approaches, and algorithms. This was the reason for Triguero et al. [9] to propose a PG taxonomy that is used to enhance *k*NN drawbacks, which was defined as a hierarchical way of three levels (generation mechanisms, resulting generation set, and type of reduction), and also review the all algorithms of the PG from the literature (see [9] for a detailed explanation).

In the next section, we introduce a brief of Self-Organizing Maps and the approach is proposed, the combination of SOM and *k*NN.

### 2.2. A Brief Summary for the Kohonen Self-Organizing Maps

Kohonen Self-Organizing Map (SOM) is a type of neural network that consists of neurons located on a regular low-dimensional grid, usually two-dimensional (2D). Typically, the lattice of the 2D grid is either hexagonal or rectangular [24]. The SOM learning or training process is an iterative algorithm which aims to represent a distribution of the input pattern objects in that regular grid of neurons. The similar input patterns are associated in the same neurons or in the adjacent neurons of the grid.

For the SOM training, a dataset is chosen and divided into two distinct sets. The training set is used to train the SOM which is here called **X**_train_. The other set is used to test the trained SOM (**X**_test_). After this dataset division, we start the training SOM. Formally, an object is randomly selected from **X**_train_ during a training, defined as **x**_*n*_ = [*x*_*n*1_, *x*_*n*2_,…, *x*_*nm*_], where the element *x*_*nm*_ is an attribute or feature of the object, which belongs to *R*^*m*^. The object is similar to what was before defined, but without the class *x*_*ny*_ information. Additionally, each neuron *j* of the SOM grid has a weight vector **w**_*j*_ = [*w*_*j*1_, *w*_*j*2_,…,*w*_*jm*_]^*T*^, where *j* = 1,2,…, *l*; here *l* is the total number of neurons of the map.

During the learning process, the input pattern is randomly selected from the training set and it is compared with the weights vector of the map, initially initialized randomly. The comparison between **x**_*n*_ and **w**_*j*_ is usually made through Euclidean distance. The shortest distance indicates the closest neuron *c*, which will have its weight vector **w**_*c*_, updated to get close to the selected input pattern **x**_*n*_. Formally, neuron *c* is defined as follows:

(1) $$\begin{array}{c} c=\underset{j}{\arg\min}\left\|\;x_{n}-w_{j}\;\right\|. \end{array}$$

The closest weights vector **w**_*c*_ and their neighbors are updated using the Kohonen algorithm [24]. However, the topological neighborhood is defined so that the farther away the neuron from **w**_*c*_, the lower the intensity for the neighborhood to be updated. The intensity of the neighborhood function is defined in relation to the training time. In other words, in initial times, the level has high value and, according to the next iterations, it is reduced at each iteration. See Kohonen [24] for a complete explanation of the training rule of the SOM map.

### 2.3. Building a Prototype Generation Based on SOM

Since the training phase has been completed, each input pattern object from the training set has to be grouped to the closest neuron. The idea in this approach of using SOM as a PG technique is that the index of each instance **x**_*n*_ is a part of the nearest neuron list. Thus, the list of each neuron *j* is here called the Best Matching Unit List (BMUL), formally defined as

(2) $$\begin{array}{c} \text{BMU}{\text{L}}_{j}=\left\{\;n\;|\;d\left(\;x_{n},w_{j}\;\right)\leq d\left(\;x_{n},w_{i}\;\right)\vee i\neq j\;\right\}, \end{array}$$

where *j* is assigned to the number of the map neuron and BMUL is a list with the indexes *n* of input patterns objects associated with the nearest neuron.

The relationship between the instance of training set **x**_*n*_ and the list of the best match unit BMUL_*j*_ is of many-to-one. That is, some units *j*, which we could call microclusters, must be associated with one or more instances and other units may have no associations; that is, the list can be empty {*∅*}.

**Figure 1:**
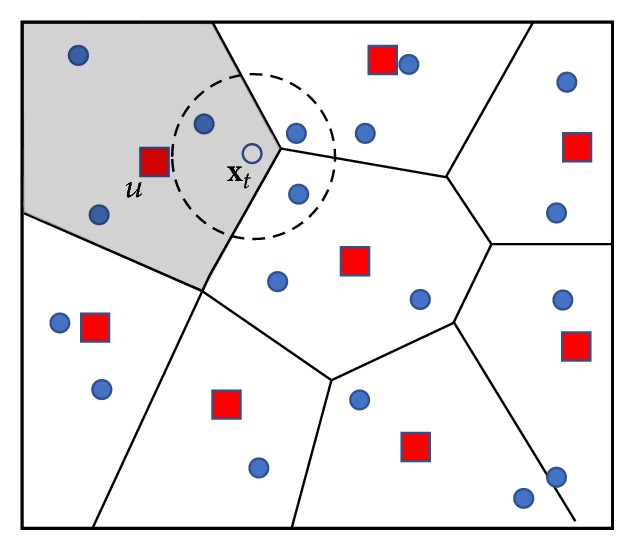
The border between the weight vectors (squares) can be interpreted as a Voronoi region (shaded area). Thus, the input patterns object (filled circles) belongs to a Voronoi region.

The classification method proposed herein explores two important characteristics of the SOM: vector quantization and topological ordering [24]. For better understanding these features, consider the representation of Figure 1 with input patterns objects (filled circles) used for training a SOM map and the weight vectors of each neuron (squares) after the training phase. In this figure, each weight vector represents a microcluster of input patterns, which is a quantization characteristic. The relationship between the weight vectors can be interpreted as a boundary, which can be understood as a Voronoi region, as exemplified by the shaded area in Figure 1. In operational aspects of use, this can be considered in a classification process in which the strategy, introduced and explored herein, means to establish a two-step process. In the first step, when a test sample **x**_*t*_ (see Figure 1, the unfilled circle) is compared to the weight vectors of the trained SOM map (the squares of Figure 1), the algorithm defines the closest unit *u* according to the following equation:

(3) $$\begin{array}{c} u=\underset{j}{\arg\min}\left\|\;x_{t}-w_{j}\;\right\|. \end{array}$$

Hence, as *u* is the nearest unit, we know the list with input patterns indices that should be queried, that is, BMUL_*u*_. Illustratively, consider that weight vector *u* belongs to a Voronoi region; see Figure 1, the shaded area, which has a BMUL_*u*_ list with the indices of input patterns known (filled circle). Also in this figure, the unlabeled sample **x**_*t*_ (unfilled circle) belongs to the region covered by unit *u* (shaded area); that is, in the second step of the classification process, the *k*NN algorithm is performed with a reduced set of objects.

However, note that the input patterns object stored in the dataset (filled circles), which are the closest to the object being classified **x**_*t*_ (unfilled circle), belong to neighboring Voronoi regions and are consequently represented in other lists; see Figure 1, circle with a dotted line.

For that reason, in a classification task with *k*NN or (*i*NN as will be introduced in the next section) combined with SOM, the use of the objects represented only as BMUL_*u*_ list results in a substantially reduced classification process time but can reduce the accuracy rate. Thus, we explored the second important feature of SOM, the topological ordering of the training dataset objects. In other words, in addition to the BMUL_*u*_ list, the lists of adjacent neurons in the SOM map grid are also consulted.

The visit of adjacent units depends on the grid initially set at the SOM training phase. For the SOM trained with rectangular lattice topology, the units of the four adjacent units should be considered. Thus, the query list BMUL_query_ for the unknown pattern **x**_*t*_ is defined as

(4) BMULquery=BMULu,BMULutop,BMULuright,BMULubottom,BMULuleft.

Otherwise, for a hexagonal lattice topology, we have to consider six adjacent units and so on. In previous studies using SOM with *k*NN [5, 20, 21], we compared the two neighborhood topologies (rectangular and hexagonal) and the results were equivalent. For this reason, the rectangular lattice topology was chosen in this work.

Finally, in the second step of the classification method proposed here, the reduced objects set belonging to BMUL_query_ (4) is used to find the *k* nearest neighbors (*k*NN). Note that the set of objects extracted from the query lists, that is, **X**_BMUL_query__, is part of the set of input patterns objects used for the SOM training; that is, **X**_train_ = [**x**_1_, **x**_2_,…,**x**_*n*_]^*T*^ and **X**_BMUL_query__ ⊂ **X**_train_. Formally, we have

(5) $$\begin{array}{c} X_{\text{BMU}{\text{L}}_{\text{query}}}=\left\{\;x_{\text{bmul}}\;|\;\text{bmul}\in \text{BMU}{\text{L}}_{\text{query}}\;\right\}. \end{array}$$

Thus, the class of the *k* nearest (or *i* informative instances as will be explained in the next section) is used to label the unknown sample **x**_*t*_. This framework combination was initially called SOM*k*NN (and here will be introduced the SOM*i*NN classifier).

In summary, the conventional algorithm NN (or *k*NN) compares the unknown sample with all the instances of the dataset; here, the comparison is limited to a selection of the objects; that is, the comparison is restricted to a small number of instances from the training dataset. The main implementation steps are described as a pseudo-code in Algorithm 1.

As verified in this section, we formalized a strategy to select input pattern objects to be used as references in a classification task and to speed the time of *k*NN algorithm. The next section introduces the *i*NN algorithm which is less sensible to *k* parameter and for this works better than *k*NN in datasets with overlapped classes (boundary not well defined).

### 2.4. Informative Nearest Neighbors

Some data classification approaches based on nearest neighbor, in addition to defining a given range of *k* values to find the nearest neighbors, also utilize new distance metrics, such as the informative nearest neighbor [10]. In other words, they utilize in the analysis of a new object of unknown class a measure that quantifies which training set object is most informative.

In order to find the informative nearest neighbor, the *i*NN algorithm, as it is called in the proposal by Song et al. [10], calculates the informativity through the following equation:

(6) Ixi ∣ xt=−log⁡1−Pxi ∣ xt×Pxi ∣ xt,i=1,…,N,

where *I* is the value of the informativity between the neighbor **x**_*i*_ and the object under analysis of unknown class (**x**_*t*_), to the extent that *P*(**x**_*i*_∣**x**_*t*_) is the probability of the object **x**_*i*_ being the informative nearest neighbor. This probability is defined by the following equation:

(7) Pxi ∣ xt=Prxi ∣ xtη∏n=1N1−Prxi ∣ xt×⨿ci¬cn.

The first term in (7) Pr(**x**_*i*_∣**x**_*t*_)^*η*^ is defined as the probability that the object **x**_*i*_ is close to the object **x**_*t*_ and *η* is defined as *N*_**x**_*i*__/*N*, where *N*_**x**_*i*__ is the number of objects that have the same class as **x**_*i*_. The second part in (7) indicates the probability that the object **x**_*i*_ is distant from the other objects of the training dataset **x**_*n*_. The indicator ⨿[·] will be 1 if the class attributes of the objects **x**_*i*_ and **x**_*n*_ are different; in other words, *c*_*i*_¬*c*_*n*_. Therefore, it can be understood as a penalty factor.

The probability Pr(**x**_*i*_∣**x**_*t*_) in (7) can be defined as a function of distance between the objects; in other words,

(8) $$\begin{array}{c} Pr\left(\;x_{i}\;|\;x_{t}\;\right)=\exp\left(\;-{\left\|\;x_{i}-x_{t}\;\right\|}^{2}\;\right). \end{array}$$

**Figure 2:**
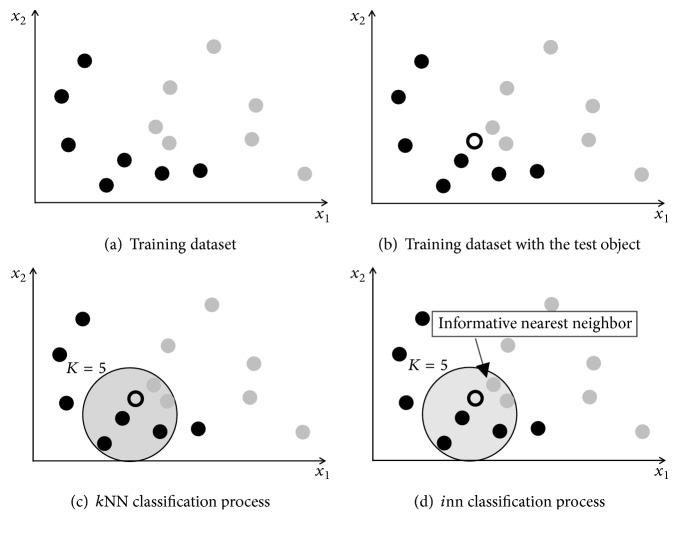
Illustration of the classification process made by the *k*NN and *i*NN algorithm.

To understand the *i*NN algorithm in practical terms, consider the dataset utilized in Figure 2(a), where it is represented by shaded circles, to the extent that the shades (dark and light) represent the two classes of the set. Now consider Figure 2(b), which has the same training objects with the addition of an object without class represented by a circle without shade. Now, consider in Figure 2(c) the contours in training objects and test object, representing the classification process executed utilizing the traditional *k*NN, with Euclidean distance and *k* value being equal to 5. In this process, the majority class of the nearest neighbors is the one that is represented by dark shading. And, therefore, the decision-making process is made by this class. However, the object under analysis has as its nearest neighbor an object of the training set that belongs to the class with light shading and this, on the other hand, also has as its neighbor another object of the same class. Therefore, utilizing the *i*NN algorithm, the informativity takes into consideration not only the majority class but also the nearest objects and the concordance that the other objects of the training set have with the nearest object. In conclusion, in the case of the *i*NN, the classification would be made by the class represented by the lightest shading Figure 2(d).

Thus, the concept of informative nearest neighbor has the following definitions. Within the *k* nearest neighbors, the object that is nearest to the object that is being classified, which is distant from other objects of different classes, is considered the most informative object, such that its class is attributed to the unknown object. On the other hand, the object that has a different class from the most informative object is considered least informative. An object is also considered least informative within the *k* nearest neighbors when it has the same class as the most informative object and is nearest to other objects of different classes.

The informativity calculation has a high computational cost because, in addition to comparing the object under analysis with the objects of the training set (first part in (7)), the algorithm still requires a comparison between the training set objects (second part in (7)). In order to reduce the computational effort, Song et al. [10] suggest having the execution of the *k*NN algorithm before executing the *i*NN to define a reduced dataset with *k* most similar objects, according to the Euclidean metric. However, the *k*NN algorithm has the disadvantages presented in the Introduction (the need to store the training set, noise sensibility, etc.) and its use before the *i*NN can affect the performance in the classification of objects that are in a border region, as illustrated in Figure 2(c).

The following section presents a proposal that combines the SOM with the *i*NN algorithm to build a process that will be named SOM*i*NN. This section will also show the advantages of the SOM*i*NN over the *i*NN.

## 3. Methodology for Combining SOM and *i*NN: A Hybrid Classification SOM*i*NN

The approach utilized by the SOM*i*NN classifier explores the concept of quantization, topology maintenance, and informativity. As already mentioned, an informative object allows the correct prediction of an unknown object, even in boundary not well defined. When talking about information, we cannot have information quality without first significantly measuring this. Information quality is one of the determining keys for the quality of the decisions and actions that are made according to it [25]. It is exactly what the SOM*i*NN classifier proposes to do; in other words, before predicting the class of the unknown object, it measures the information of the training set objects before making the classification decision.

**Figure 3:**
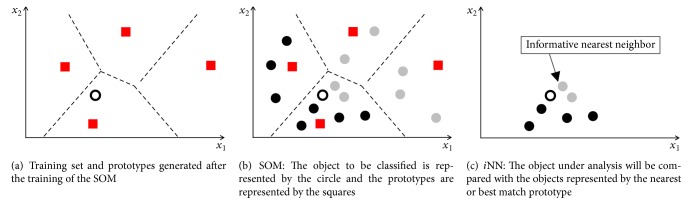
Illustration of the classification process made by the SOM and *i*NN.

In order to understand the SOM*i*NN combination, consider a SOM trained with the objects from Figure 2(a) without using the class information (shaded color). The prototypes adjusted resulting in trained SOM map (weight vectors) are represented in Figure 3(a). The result of the SOM can be generally understood as being a summary of the training set, through a set of prototypes that have a Voronoi region, with the number of prototypes being smaller than that of the training set, in the following example: the twelve objects were summarized into four prototypes. The number of prototypes is a parameter that refers to the number of neurons of the SOM map.

Now consider the new object classification submitted to process that was presented in Figure 2(b). Also consider the prototype set being utilized in a first comparison, instead of the training set. In this case, for the classification process, in initial phase a comparison will be done between the object under analysis and the set of prototypes, as illustrated in Figure 3(a). Repeating the process that takes place in training the SOM for the selection of the winning neuron, made using the Euclidean distance, the nearest neuron is selected (winner or best match) to the object under analysis. From this process where the nearest prototype is known and that, on the other hand, it is possible to know which training set objects are represented by the prototype, see Figure 3(b) where each prototypes has a Voronoi region. Thus, the reduced training set objects are retrieved to start the classification phase with the *i*NN. Finally, the classification will be done with a reduced set, as shown in Figure 3(c).

In summary, the last step in Algorithm 1, the use of classifier algorithm, is executed with *i*NN. This process is called here as SOM*i*NN classifier.

Since the process will depend on the selection of the number of neurons of the SOM map, we will utilize the empirical proposal of Vesanto et al. [26] that defines the number of neurons as being the root of the number of objects of the training set. What happens is that, after training the SOM map, some prototypes can be empty; in other words, the prototype represents no object of the training set. In order to prevent this situation from happening, the proposal of Silva and Del-Moral-Hernandez [5] will be utilized. Thus, besides retrieving the objects of the winning prototype, it will also consider the retrieval of the adjacent prototypes.

The combination using the SOM neural networks approach with the *i*NN explores the main characteristics that define the potential of a data classifier, which are storage reduction, noise tolerance, generalization accuracy, and time requirements [9]. To the contrary of the *i*NN that preprocesses the data utilizing the *k*NN algorithm that has a high computational cost, the algorithm proposed in this work reduces the data representation through the SOM. In addition, with the use of the SOM, the classification time of the SOM*i*NN is expected to be shorter when compared with the *i*NN, which results in less memory use, maximizing the classifier's performance in terms of classification time.

The next section highlights all steps that were done to make the experiments with the SOM*i*NN classifier.

## 4. Experimental Methodology, Results, and Analysis

This section will present the dataset utilized in the experiments and the parameterization of the classifiers utilized for the comparison with our SOM*i*NN proposal. The experiments consist in using an artificial dataset for qualitative and quantitative analysis and with public dataset used as benchmarking in the literature to evaluate the efficacy of the algorithm proposed.

### 4.1. Datasets

In order to provide a qualitative analysis with visualization of the border decision-making area and a quantitative analysis in terms of classifier accuracy, three datasets were generated with the following features: 300 objects, two attributes, two classes, and a balanced number of objects per class. For all datasets, the objects were distributed with the same mean but with difference in the standard deviation value, in order to force an overlapping of classes. Thus, each dataset represents distinct situations on the border of classes: no, low, and high confusion.

In order to evaluate the efficacy of the algorithm proposed and compare it with others from the literature, 21 public databases were chosen (Repository of the University of California, Irvine, UCI) that are used as benchmarking for Prototype Generation approaches.  Table 1 summarizes the properties of each benchmarking dataset in number of objects (Obj), number of attributes (Att), and number of classes (Cla). For all databases, the attributes are numerical. The separation of these datasets in training and test set were done with the use of the 5-fold cross validation.

### 4.2. Parameterization of Algorithms

The SOM*i*NN approach will be compared with *k*NN, *i*NN, and SOM*k*NN. The classifiers parameterizations are represented in Table 2. The SOM parametrization is the same for SOM*k*NN and SOM*i*NN.

The experiments were implemented using the R language, version 3.1.2, with RStudio IDE version 0.98 and using a conventional computer with Windows 10, i7 with 8 GB RAM. The experimental results are presented in the following section.

### 4.3. Qualitative and Quantitative Analysis Using the Artificial Dataset

**Figure 4:**
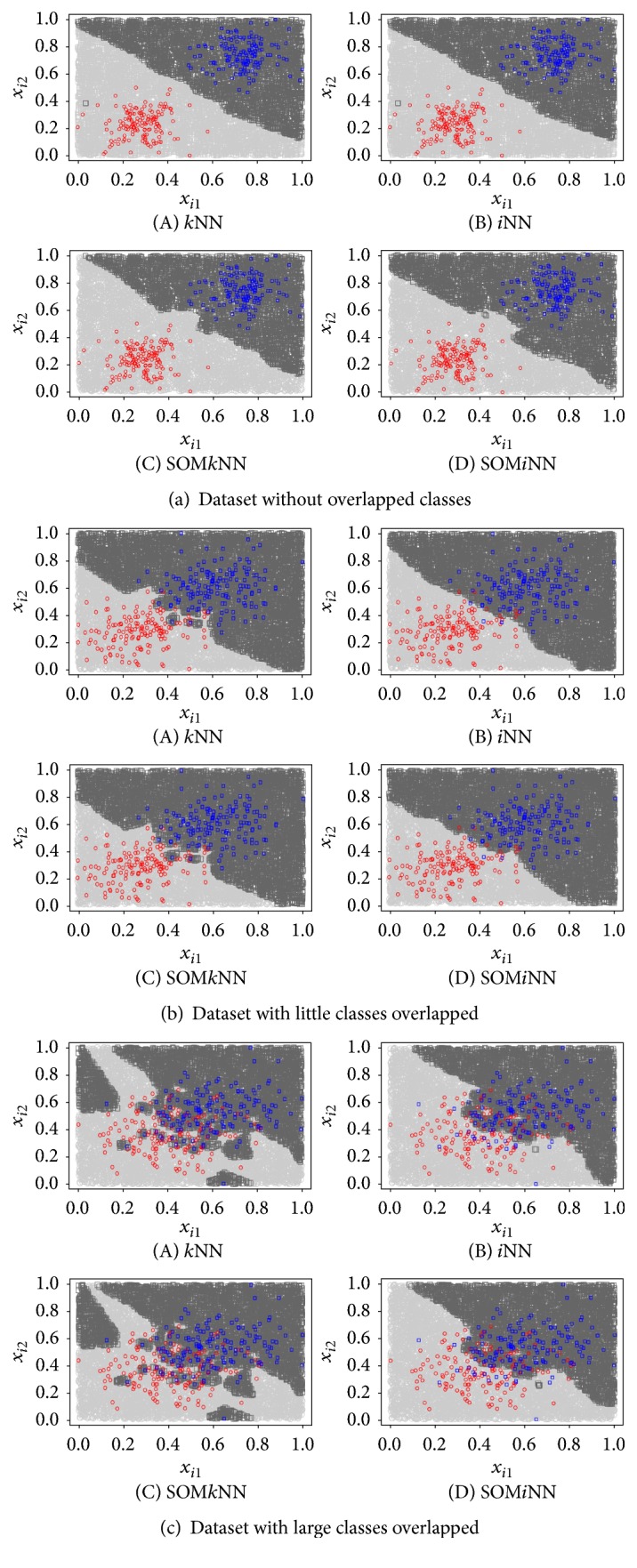
For each experiment, a set of four results was conducted and the results are (A) *k*NN; (B) *i*NN; (C) SOM*k*NN; and (D) SOM*i*NN.

The objective of experiments using artificial dataset was to compare the performance of *i*NN, *k*NN, SOM*i*NN, and SOM*k*NN classifiers in situations where there are well-separated classes (Figure 4(a)), classes partially overlapped (Figure 4(b)), and a large number of classes overlapped (Figure 4(c)).

Analyzing qualitatively, starting by Figures 4(a)(A), 4(b)(A), and 4(c)(A),  *k*NN results, we can note that the boundary separation degrades from the moment that the classes start the overlapping. In the worst case, we can observe that a high overlap (Figure 4(c)) is clearly one of the *k*NN disadvantages, because it makes the decision boundary considering all objects as having the same importance. For the *i*NN results (Figures 4(a)(B), 4(b)(B), and 4(c)(B)), it is clear that the border of separation is softer, even when the class overlap increases. This is because the separation was defined by informative representation of the objects from the same class. This fact is most evident in the last experiment (Figure 4(c)), where we can observe that the boundary separation is created to preserve the predominant class in the border region.

Figures 4(a)(C and D), 4(b)(C and D), and 4(c)(C and D) are the results using SOM as the Generation Prototype approach. That is, the decision boundary was generated without using all objects of the database but, instead, based on objects distributed in prototypes of the trained SOM map. In this qualitative analysis, the most important to note is that the preservation of the decision boundary was maintained in all experiments, without significant changes.

**Figure 5:**
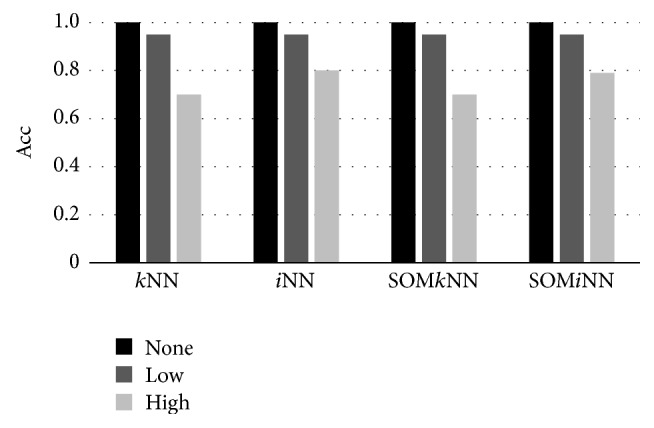
Accuracy results for classifiers using artificial dataset.

Finally, we analyzed quantitatively the experiments with artificial data, with an average classification accuracy defined by a dataset with 10^6^ objects (which were used to generate the decision boundary of Figure 4). The results are shown in Figure 5. The conclusion for the experiments using artificial dataset is that the use of *i*NN is more effective than *k*NN when the separation class has high confusion and that for this, the performance accuracy has not been abruptly reduced. We also note in this qualitative analysis that the use of SOM as Prototype Generation method does not significantly degrade the accuracy performance.

The use of artificial datasets can make qualitative and quantitative analyses between the classifiers. The next experiment has the objective of expanding the previous study [23] through analysis with other performance measures, such as kappa, impact of dataset reduction on the accuracy, and performance of classification time. For these new experiments, 12 new public datasets were used that are benchmarking in Prototype Generation approach [9].

### 4.4. Experiments and Analysis Using the Benchmarking Dataset

This section shows the experiments and results for datasets introduced in Table 1. The results are analyzed using the following measures as performances: accuracy, kappa, hypothesis test, rate of dataset reduction, and classification time.

Table 3 shows all the classification results for the paper experiments. In this table, the accuracy and kappa measures are shown in terms of average and standard deviation. The other results are also discussed in this section.

In practical aspects, the accuracy and kappa measures are equivalent in terms of performance. For purposes of simplification, only the accuracy will be considered in the extended discussion of the result analysis.

The accuracy is analyzed by comparing the results of the classifiers in pairs. The average and the result deviation of each dataset are compared using the *t*-test with 95% of confidence interval. The comparison result is shown in Table 4. In this table, the datasets indices (“#,” see Table 1) are separated according to the classifier results: higher results (*X* > *Y*), equal results (*X* = *Y*), and lower results (*X* < *Y*), with *X* and *Y* being a representation of classifiers compared in pairs. From this result, the same comparison structure will be used again for the count of the incidences percentage.

The general counting is shown in Table 5. This table has an additional column to represent the sum of the percentages of equal and lower results, in order to show when a classifier performance is really better than the other. This was the reason to compare (*X* > *Y*) with (*X* = *Y*) and (*X* < *Y*) in Table 5.

Analyzing the results from Table 5 is possible to note in the first two lines of table that *i*NN showed to be better than *k*NN in most of cases (66.7% and 52.4%, combined with SOM). The use of SOM in the classification process (last two lines of the table) has been shown to be slightly better or worse in some cases. The SOM performance with *i*NN is improved (52.4%) and with *k*NN there is a little degradation (47.7%). However, an important result that should be emphasized (last row of the table) is that the use of SOM with *i*NN maintains or improves the performance in most databases (52.4%).

**Figure 6:**
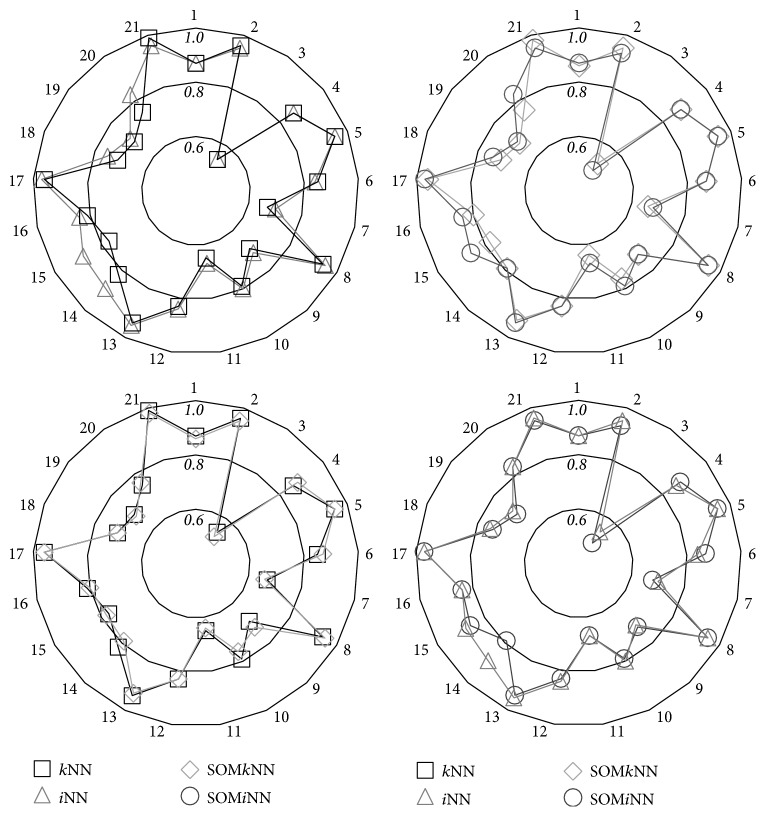
Radar graphic contrasting pairs of classifiers.

As a final analysis of the accuracy performance, in order to show that the degradation with the use of SOM has little impact on the final performance, the comparison of the same pair of classifier presented above is performed using a radar chart (Figure 6). In this graph, the external values (polar scale) indicate the dataset number of 1 to 21, and the internal values show the accuracy performance, starting from 0.6 to 1.0. The ideal result would be to have the graph contour in 1.0. In this study, the main results are obtained for the overlapped lines, representing an equivalent result for contrasted classifiers. Combining the results of the accuracy performance (statistical and chart), we can consider that *i*NN has, in the vast majority of studies, a superiority in the classification performance when compared to *k*NN. From this result, it is interesting to note that the *i*NN superiority occurs mainly in datasets with performance below 90% as follows: 7, 9, 11, 12, 14, 15, 16, 18, 19, and 20. On the other hand, the result is lower in experiments with datasets 2 and 21, that is, where the accuracy performance is close to 1 (100%). Therefore, the results also suggest that *i*NN has superior results in datasets in which the decision boundary is not well separated.

**Figure 7:**
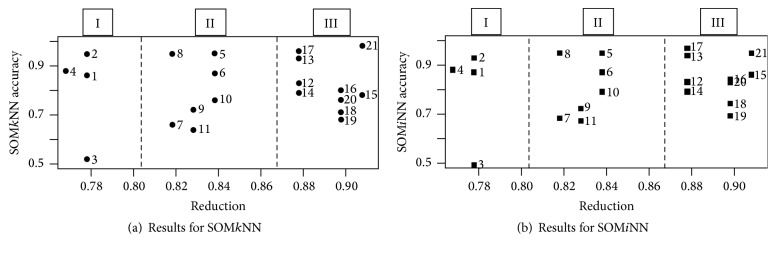
Results of reduction per accuracy.

The next analysis consists in verifying the SOM efficiency in reducing input objects. For this, the reduction and accuracy percentage of each dataset performance is checked. The results are shown in Figure 7. Interestingly, in both results, SOM*k*NN and SOM*i*NN, there are three regions very well defined in the accuracy reduction experiment. The first datasets have an average of 150.5 objects, with the second averaging 215 and the last averaging 694.5 objects. That is, the reduction varies with the number of objects. Therefore, the results of SOM*k*NN (Figure 7(a)) and SOM*i*NN (Figure 7(b)) show that the more objects in dataset, the higher the reduction rate.

**Figure 8:**
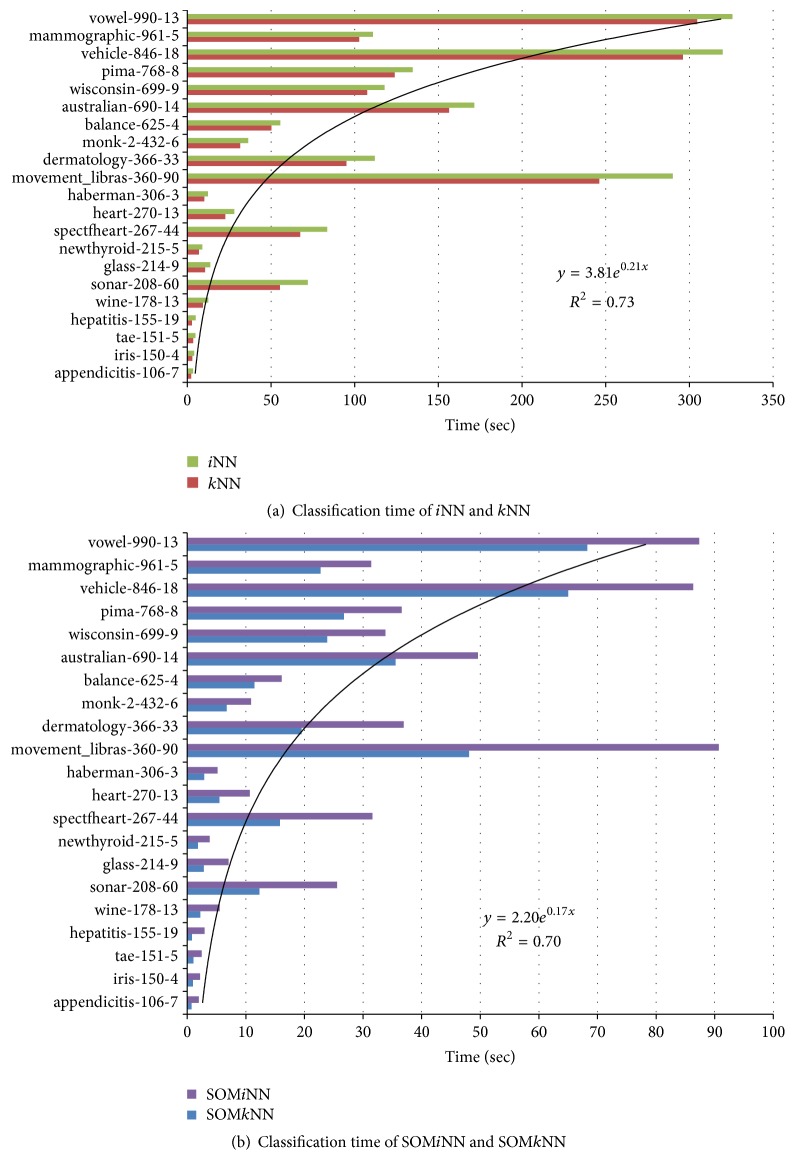
Classification time analysis. The datasets are organized in ascendant order of number of objects.

The next results to be analyzed are the time consumed in the classification process. The results are shown in Figure 8, and, for interpretation purposes, the databases are arranged in the vertical axis and are organized in ascending order of number of objects. In vertical axis, each dataset is described by name, number of objects, and number of attributes (described in Table 3). The time shown on the horizontal axis is measured in seconds.

By analyzing in detail the result of the time classification algorithms *i*NN and *k*NN in Figure 8(a), it is observed that, to a certain number of objects, around 180 (datasets appendicitis to wine), the classification time is almost linear. From this point the tendency curve is not clear. The reason is that there are an increasing number of objects in these other databases and also a variation in the number of attributes. This means that the classification time depends not only on the number of objects but also on the number of attributes, for example, the balance database (625 objects) and dermatology (366 objects), whose last dataset has a smaller number of objects and consumes more time. Another interesting case to mention is observed between the base* mov_libras* and* vowel*. The former has almost half the number of objects and nearly ninefold more attributes than the latter but both consumed an equivalent time in the classification process. Another point to consider in the graph is that, for every experiment, the classification time of *i*NN is higher than *k*NN. This result was expected because, as mentioned earlier, *i*NN is computationally more costly due to the fact that *k*NN is run before it as a preprocessing step and, thus, it finds the closest informative object. Although it seems to be an obvious result, the experiments confirm their reliability. Finally, for a general idea of the time, a tendency line was added to the results and the best adjustment was an exponential trend, with Pearson coefficient above 0.7, which is considered a high value. As it is difficult to find a relationship between the numbers of objects and attributes to explain the process timing, the trend is more indicative about the number of objects. Thus, for this experiment, the classification time is more sensitive to the number of objects.

The same time experiment discussed above was repeated for SOM*i*NN and SOM*k*NN (Figure 8(b)). The behavior of the results in this experiment is similar to that discussed for Figure 8(a). This can be interpreted in two ways. The first is that the above analysis can be applied for these results and, more importantly, that the objects selected by the reduced set SOM prototypes can maintain the characteristics of the raw database. However, it should be noted in the result analysis that the time classification scale (horizontal axis) ranges from 0 to 100 seconds. In the earlier results, the scale ranged from 0 to 350 seconds. Nonetheless, the importance of this result is that the trend remains exponential, *R*^2^ with 0.7. It is noteworthy that, in the result time shown for SOM (SOM*k*NN and SOM*i*NN), the training time is included.

**Figure 9:**
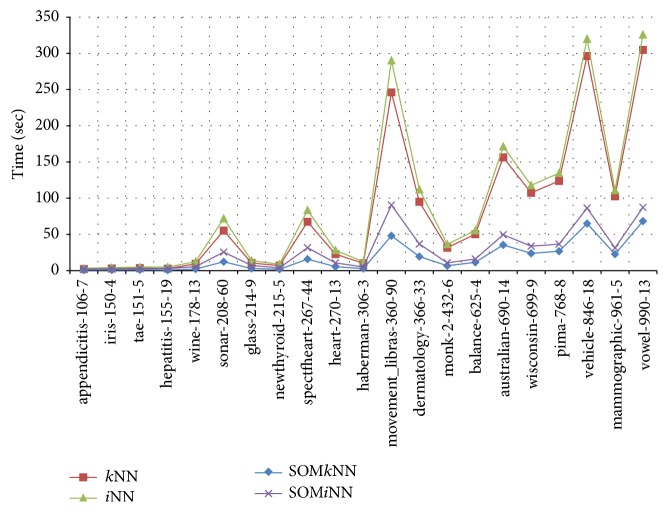
Classification time summarized for all classifiers.

For a global analysis, in the Figure 9, there are all classification time results together: *k*NN, *i*NN, SOM*k*NN, and SOM*i*NN. The databases in the horizontal axis were arranged again in quantities of objects. By analyzing qualitatively the results shown in this figure, we can note that when the number is lower than about 180 (to the* wine* dataset), the use of SOM as a preprocessing to *i*NN and *k*NN algorithms in order to reduce the time classification does not have significant advantages. Thus, the use of SOM to decrease the classification time of *i*NN and *k*NN algorithms seems to be more advantageous in database with more than 180 objects (from* sonar* dataset). This result can be observed at the upper end, where the consumption of classification time is high (*vowel* dataset), and the use of SOM can reduce by more than 3 times the *k*NN and *i*NN classification time.

### 4.5. Contrasting the Results of This Work with That in the Literature

For an idea about the importance of the results herein mainly using the *i*NN and the combination SOM*i*NN approach, the performance indexes obtained here was compared with the literature result [9]. The approach chosen for the comparative experiments, Chen algorithm, belongs to the same Prototype Generation category of SOM. The algorithm named Chen [9, 27] was executed using the datasets of Table 3 and the compiled results for this algorithm are shown in terms of average and standard deviation of accuracy, time, and reduction. The comparative results are summarized in Table 6.

Note from the comparative results of Table 6 that *i*NN is the algorithm that has the best performance accuracy. This is an important result because it is the algorithm introduced here as an alternative to *k*NN. In terms of time, the lowest result was obtained by the SOM*k*NN; therefore, it involves the SOM as the approach of Prototype Generation method introduced in this work and it is expected that *i*NN is more time consuming than the *k*NN, as discussed in Section 2. Finally, the Chen algorithm has the bigger reduction, which is to be expected too, since according to Triguero et al. [9], the prototypes parameter has to be configured as being 90% of the number of objects of the dataset.

## 5. Conclusion

This paper introduces a new classifier named SOM*i*NN, which is based on the combination of Self-Organizing Maps (SOM) and* informative* nearest neighbors (*i*NN). The *i*NN classifier is costly in computational terms, because in a classification process the informativity is not calculated only by the object under classification analysis, but also considering the other objects of the training set. Song et al. [10] suggested the use of *k*NN algorithm (with the best *k* value experimentally found as being 7) before *i*NN to minimize the high computational cost, that is, using 7-NN to find a reduced subset for the classification process with the informative nearest neighbor algorithm.

In order to contribute to the Song et al. [10], in this paper, the *k*NN has been substituted by SOM because of quantization vector and maintenance topological of raw dataset. In other words, a SOM map is trained with the dataset and, after this, the objects of this set are associated with the nearest (or winning) neurons. And, thus, each neuron of the map or prototype represents an object subset. Now, in a classification process, the object is compared with the map prototypes, where the winner is elected. The objects mapped in this winning neuron and adjacent neurons are retrieved and presented to then have the execution of *i*NN.

Thus, due to the preprocessing made by the SOM to the *i*NN algorithm, the computational effort as a whole to find the informative nearest neighbor is much smaller, which results in a significant reduction in the classification time when compared to the classification time of the *i*NN without preprocessing.

Therefore, the primary objective of the classifier addressed in this paper was the maintenance of the accuracy of the *i*NN and the reduction of the classification time in a classification process, thus concluding that the use of the objects represented by the winning neuron and adjacent neurons was effective in the analytical aspects by not degrading the performance of *i*NN. The results presented in Section 4 indicate this reduction of the time and, in addition, that the classification rates of the SOM*i*NN are statistically similar when compared to the *i*NN, that is, time reduction and accuracy preservation.

Another important conclusion in analysis of the classification experiments, mainly using artificial dataset, and also in benchmarking dataset where the accuracy performance was worst, the *i*NN approach presents more significant accuracy results when the objects of different classes are not well separated, with high mixture in the border region.

As a final conclusion, the *i*NN is an algorithm with accuracy performance better than *k*NN. But the classification time is a bottleneck for the algorithm, which is minimized using SOM as a Prototype Generation technique. Thus, the SOM*i*NN classifier is proposed here which is specialized to solve problems where the border region is not well defined in a tolerable time.

## Figures and Tables

**Algorithm 1:**
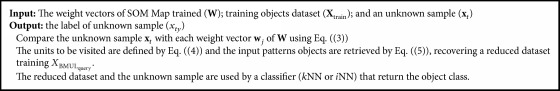
Prototype Generation based on SOM briefly described in a pseudo-code.

**Table 1:** Properties of dataset used in experimental analysis of this work. These datasets are available in UCI webpage (https://archive.ics.uci.edu/ml/datasets.html) and also in webpage auxiliar of Triguero et al. publication [9].

#	Name	Properties		
		(#Obj)	(#Att)	(#Cla)
1	appendicitis	106	7	1
2	iris	150	4	2
3	australian	690	14	16
4	balance	625	4	15
5	dermatology	366	33	13
6	glass	214	9	7
7	haberman	306	3	11
8	heart	270	13	10
9	hepatitis	155	19	4
10	mammographic	961	5	20
11	monk-2	432	6	14
12	movement_libras	360	90	12
13	newthyroid	215	5	8
14	pima	768	8	18
15	sonar	208	60	6
16	spectfheart	267	44	9
17	tae	151	5	3
18	vehicle	846	18	19
19	vowel	990	13	21
20	wine	178	13	5
21	wisconsin	699	9	17

**Table 2:** Parametrization of algorithms.

Algorithm	Parameters
*k*NN	*k* = 1 and Euclidian distance

SOM	Euclidian distance, batch training, maximum training time equal to 1000, rectangular lattice, and Gaussian neighborhood function with maximum aperture of 1 with decay due to the number of iterations. The SOM map dimension has the square root of the number of dataset objects by two ($\sqrt{N}/2$)

*i*NN	Execution of the *k*NN algorithm with *k* value equal to 7 (best result from [10]) and informative neighbor number equal to 1

**Table 3:** Classification results for all algorithms represented by mean and standard deviation of accuracy and kappa measures.

	Classifiers	Acc	Kappa
1	*k*NN	0.87 ± 0.1	0.6 ± 0.32
	*I*NN	0.87 ± 0.06	0.52 ± 0.24
	SOM-*k*NN	0.86 ± 0.09	0.57 ± 0.3
	SOM-*I*NN	0.87 ± 0.06	0.52 ± 0.24

2	*k*NN	0.96 ± 0.04	0.94 ± 0.07
	*I*NN	0.95 ± 0.05	0.93 ± 0.08
	SOM-*k*NN	0.95 ± 0.02	0.93 ± 0.03
	SOM-*I*NN	0.93 ± 0.05	0.90 ± 0.08

3	*k*NN	0.54 ± 0.1	0.30 ± 0.15
	*I*NN	0.54 ± 0.07	0.30 ± 0.10
	SOM-*k*NN	0.52 ± 0.07	0.28 ± 0.10
	SOM-*I*NN	0.49 ± 0.08	0.23 ± 0.11

4	*k*NN	0.86 ± 0.07	0.41 ± 0.32
	*I*NN	0.86 ± 0.07	0.41 ± 0.32
	SOM-*k*NN	0.88 ± 0.09	0.48 ± 0.41
	SOM-*I*NN	0.88 ± 0.09	0.48 ± 0.41

5	*k*NN	0.95 ± 0.03	0.92 ± 0.05
	*I*NN	0.95 ± 0.03	0.92 ± 0.05
	SOM-*k*NN	0.95 ± 0.03	0.93 ± 0.04
	SOM-*I*NN	0.95 ± 0.03	0.93 ± 0.04

6	*k*NN	0.85 ± 0.05	0.69 ± 0.10
	*I*NN	0.84 ± 0.04	0.68 ± 0.09
	SOM-*k*NN	0.87 ± 0.05	0.75 ± 0.11
	SOM-*I*NN	0.87 ± 0.05	0.75 ± 0.11

7	*k*NN	0.67 ± 0.05	0.55 ± 0.08
	*I*NN	0.7 ± 0.05	0.58 ± 0.07
	SOM-*k*NN	0.66 ± 0.04	0.54 ± 0.07
	SOM-*I*NN	0.68 ± 0.04	0.56 ± 0.05

8	*k*NN	0.94 ± 0.03	0.88 ± 0.05
	*I*NN	0.95 ± 0.02	0.9 ± 0.04
	SOM-*k*NN	0.95 ± 0.03	0.89 ± 0.06
	SOM-*I*NN	0.95 ± 0	0.9 ± 0

9	*k*NN	0.69 ± 0.03	0.16 ± 0.09
	*I*NN	0.71 ± 0.01	0.17 ± 0.07
	SOM-*k*NN	0.72 ± 0.05	0.2 ± 0.12
	SOM-*I*NN	0.72 ± 0.06	0.18 ± 0.12

10	*k*NN	0.79 ± 0.04	0.56 ± 0.09
	*I*NN	0.80 ± 0.03	0.59 ± 0.06
	SOM-*k*NN	0.76 ± 0.03	0.52 ± 0.06
	SOM-*I*NN	0.79 ± 0.04	0.56 ± 0.08

11	*k*NN	0.65 ± 0.05	0.11 ± 0.15
	*I*NN	0.67 ± 0.03	0.04 ± 0.02
	SOM-*k*NN	0.64 ± 0.05	0.07 ± 0.15
	SOM-*I*NN	0.67 ± 0.07	0.03 ± 0.17

12	*k*NN	0.83 ± 0.03	0.82 ± 0.03
	*I*NN	0.84 ± 0.03	0.82 ± 0.04
	SOM-*k*NN	0.83 ± 0.03	0.82 ± 0.04
	SOM-*I*NN	0.83 ± 0.03	0.82 ± 0.04

13	*k*NN	0.94 ± 0.02	0.92 ± 0.02
	*I*NN	0.95 ± 0.02	0.94 ± 0.02
	SOM-*k*NN	0.93 ± 0.02	0.92 ± 0.02
	SOM-*I*NN	0.94 ± 0.01	0.93 ± 0.01

14	*k*NN	0.82 ± 0.12	0.63 ± 0.25
	*I*NN	0.89 ± 0.04	0.78 ± 0.09
	SOM-*k*NN	0.79 ± 0.08	0.58 ± 0.15
	SOM-*I*NN	0.79 ± 0.03	0.58 ± 0.06

15	*k*NN	0.77 ± 0.02	0.6 ± 0.03
	*I*NN	0.88 ± 0.02	0.78 ± 0.03
	SOM-*k*NN	0.78 ± 0.03	0.62 ± 0.04
	SOM-*I*NN	0.86 ± 0.02	0.75 ± 0.05

16	*k*NN	0.81 ± 0.03	0.61 ± 0.06
	*I*NN	0.84 ± 0.04	0.68 ± 0.08
	SOM-*k*NN	0.8 ± 0.04	0.6 ± 0.08
	SOM-*I*NN	0.84 ± 0.04	0.68 ± 0.07

17	*k*NN	0.96 ± 0.02	0.90 ± 0.04
	*I*NN	0.97 ± 0.01	0.94 ± 0.02
	SOM-*k*NN	0.96 ± 0.02	0.92 ± 0.03
	SOM-*I*NN	0.97 ± 0.01	0.94 ± 0.02

18	*k*NN	0.71 ± 0.03	0.34 ± 0.05
	*I*NN	0.75 ± 0.02	0.42 ± 0.05
	SOM-*k*NN	0.71 ± 0.03	0.35 ± 0.06
	SOM-*I*NN	0.74 ± 0.04	0.42 ± 0.07

19	*k*NN	0.69 ± 0.02	0.58 ± 0.03
	*I*NN	0.71 ± 0.03	0.62 ± 0.04
	SOM-*k*NN	0.68 ± 0.02	0.57 ± 0.03
	SOM-*I*NN	0.69 ± 0.04	0.59 ± 0.05

20	*k*NN	0.75 ± 0.02	0.51 ± 0.04
	*I*NN	0.83 ± 0.02	0.66 ± 0.03
	SOM-*k*NN	0.76 ± 0.01	0.52 ± 0.03
	SOM-*I*NN	0.83 ± 0.02	0.65 ± 0.04

21	*k*NN	0.99 ± 0.01	0.99 ± 0.01
	*I*NN	0.96 ± 0.01	0.96 ± 0.01
	SOM-*k*NN	0.98 ± 0.01	0.98 ± 0.01
	SOM-*I*NN	0.95 ± 0.01	0.95 ± 0.01

**Table 4:** Classifiers compared in pairs and datasets index (“#”) where the performance is significantly improved.

*X*	*Y*	*X* > *Y*	*X* = *Y*	*X* < *Y*
*i*NN	*k*NN	7,8, 9,10,11,12,13,14,15,16,17,18,19,20	1,3, 4,5	2,6, 21
SOM*i*NN	SOM*k*NN	1,7, 10,11,13,15,16,17,18,19,20	4,5, 6,8, 9,12,14	2,3, 21
*k*NN	SOM*k*NN	1,2, 3,7, 10,11,13,14,16,19,21	5,12,17,18	4,6, 8,9, 15,20
*i*NN	SOM*i*NN	3,7, 10,12,13,14,15,18,19,21	1,2, 5,8, 11,16,17,20	4,6, 9

**Table 5:** Dataset percentage for performance analysis in terms of statistical significance.

*X*	*Y*	*X* > *Y*	*X* = *Y*	*X* < *Y*	(*X* = *Y*)+(*X* < *Y*)
*i*NN	*k*NN	66.7%	19.1%	14.2%	33.30%
SOM*i*NN	SOM*k*NN	52.4%	33.3%	14.3%	47.6%
*k*NN	SOM*k*NN	52.4%	19.1%	28.62%	47.7%
*i*NN	SOM*i*NN	47.6%	38.1%	14.3%	52.4%

**Table 6:** Comparing the results of this work with the Chen algorithm [27].

	Accuracy	Time	Reduction
*k*NN	0.81 ± 0.04	88.04 ± 0.05	0
*i*NN	0.83 ± 0.03	99.04 ± 107.81	0
SOM*k*NN	0.81 ± 0.04	19.32 ± 21.26	0.85 ± 0.05
SOM*i*NN	0.82 ± 0.04	29.76 ± 30.68	0.85 ± 0.05
Chen	0.79 ± 0.01	30.32 ± 31.83	0.87 ± 0.10
